# Assessment of Water and Nitrogen Use Efficiencies Through UAV-Based Multispectral Phenotyping in Winter Wheat

**DOI:** 10.3389/fpls.2020.00927

**Published:** 2020-06-26

**Authors:** Mengjiao Yang, Muhammad Adeel Hassan, Kaijie Xu, Chengyan Zheng, Awais Rasheed, Yong Zhang, Xiuliang Jin, Xianchun Xia, Yonggui Xiao, Zhonghu He

**Affiliations:** ^1^Institute of Crop Sciences, National Wheat Improvement Centre, Chinese Academy of Agricultural Sciences (CAAS), Beijing, China; ^2^Institute of Cotton Research, CAAS, Anyang, China; ^3^Department of Plant Science, Quaid-i-Azam University, Islamabad, Pakistan; ^4^International Maize and Wheat Improvement Centre (CIMMYT) China Office, c/o CAAS, Beijing, China; ^5^Institute of Crop Sciences, Chinese Academy of Agricultural Sciences/Key Laboratory of Crop Physiology and Ecology, Ministry of Agriculture, Beijing, China

**Keywords:** nitrogen content, phenotyping, vegetation indices, UAV, utilization efficiency, water contents, wheat

## Abstract

Unmanned aerial vehicle (UAV) based remote sensing is a promising approach for non-destructive and high-throughput assessment of crop water and nitrogen (N) efficiencies. In this study, UAV was used to evaluate two field trials using four water (T0 = 0 mm, T1 = 80 mm, T2 = 120 mm, and T3 = 160 mm), and four N (T0 = 0, T1 = 120 kg ha^–1^, T2 = 180 kg ha^–1^, and T3 = 240 kg ha^–1^) treatments, respectively, conducted on three wheat genotypes at two locations. Ground-based destructive data of water and N indictors such as biomass and N contents were also measured to validate the aerial surveillance results. Multispectral traits including red normalized difference vegetation index (RNDVI), green normalized difference vegetation index (GNDVI), normalized difference red-edge index (NDRE), red-edge chlorophyll index (RECI) and normalized green red difference index (NGRDI) were recorded using UAV as reliable replacement of destructive measurements by showing high r values up to 0.90. NGRDI was identified as the most efficient non-destructive indicator through strong prediction values ranged from *R*^2^ = 0.69 to 0.89 for water use efficiencies (WUE) calculated from biomass (WUE.BM), and *R*^2^ = 0.80 to 0.86 from grain yield (WUE.GY). RNDVI was better in predicting the phenotypic variations for N use efficiency calculated from nitrogen contents of plant samples (NUE.NC) with high *R*^2^ values ranging from 0.72 to 0.94, while NDRE was consistent in predicting both NUE.NC and NUE.GY by 0.73 to 0.84 with low root mean square errors. UAV-based remote sensing demonstrates that treatment T2 in both water 120 mm and N 180 kg ha^–1^ supply trials was most appropriate dosages for optimum uptake of water and N with high GY. Among three cultivars, Zhongmai 895 was highly efficient in WUE and NUE across the water and N treatments. Conclusively, UAV can be used to predict time-series WUE and NUE across the season for selection of elite genotypes, and to monitor crop efficiency under varying N and water dosages.

## Introduction

Low water and nutrient uptake efficiency of crops is one of the most detrimental limitation in agriculture productivity (Chuan et al., 2016; Lesk et al., 2016). Efficient irrigation and nitrogen (N) supply according to the plant requirement is a key regulator for resource-efficient crop yield (Christopher et al., 2016; Chuan et al., 2016; Nehe et al., 2018; Thorp et al., 2018). Amelioration in uptake efficiency can be adjusted by manipulation of dosage of water and N-fertilizer (Ali and Talukder, 2008) or by improving input use efficiency in crop cultivars (Hawkesford, 2017). Genetic improvement of genotypes for water and N uptake, defined as the ratio of crop yield and water or N consumption capacity of plants is an important goal in crop breeding (Passioura, 2012). Nowadays, the use of N fertilizer increased from 9.2 Mt of pure N in 1960 to 108 Mt of pure N in 2015 worldwide (FAO, 2018). Over-dosage and low N utilization efficiency of crops have caused major resources concerns and substantial greenhouse gas emissions (Guo et al., 2010; Zhang et al., 2015). In China, the agricultural system generally relies on the high-to-excessive N inputs and the total average application of N for winter wheat has increased more than 500 kg N/ha, while the nitrogen-use efficiency (NUE) in wheat system remains lowest as compared with other crops like maize and rice (Cui et al., 2018). Moreover, the traditional irrigation of winter wheat is up to 310 mm and the water use efficiency (WUE) is lower than that of the world (Sun et al., 2011). Establishing substantial regulations and breeding new varieties for high water and N acquisition could provide an effective approach to improve WUE, NUE, and yield potential (Hu and Xiong, 2014; Zhang et al., 2017).

Assessment of resource efficiencies could be detected through physiological indicators such as biomass, water status, N contents and chlorophyll level of plants (Ali and Talukder, 2008; Potgieter et al., 2017). Previously, several studies have been conducted to assess NUE and WUE through destructive approaches for selection and genetic improvement in wheat (Zhang et al., 2007; Haile et al., 2012; Hawkesford, 2017). But destructive methods are considered as bottleneck for rapid and precise estimations of biomass, N and water status at multiple time points in case of large number of genotypes (Cobb et al., 2013; Potgieter et al., 2017). Therefore, use of advance phenotyping technology can increase the precision in data collection and future decision on crop improvement (Araus and Cairns, 2014; Rasheed et al., 2020). Ground-based and aerial-based non-destructive phenotyping systems have been validated as complementary platforms for several traits like green cover, biomass, water stress severity, chlorophyll level, and photosynthesis rate (Araus and Kefauver, 2018; Hassan et al., 2018a). The unmanned aerial vehicle (UAV) platform is capable of covering larger area in a shorter period of time. This can minimize the measurement error caused by changes in environmental factors, and is independent of field condition which can disturb the movement of ground-based systems (Potgieter et al., 2017; Yang et al., 2017). UAV-based remote estimation of canopy water and N-status can provide implication on detection of physiological status for establishing decisions and immediate adopt measures for appropriate irrigation and N-fertilizer applications (Yi et al., 2013; Li et al., 2018). Previously, UAV-based multispectral and RGB imagery have been validated for detection of biomass, plant density, leaf area, senescence rate and photosynthetic activity in wheat, barley, and sorghum (Bendig et al., 2014; Sankaran et al., 2015; Jin et al., 2017; Potgieter et al., 2017; Hassan et al., 2018a, 2019).

Physiological traits such as chlorophyll content, nitrogen concentration, and water status are often hard to be assessed by the human eye but can be detected through variations in reflectance of light spectrum (Haboudane et al., 2002; Mutanga and Skidmore, 2004; Zhang and Zhou, 2019). UAV-based remote sensing has given a great opportunity to assess plants growth by capturing different bands (Blue, NIR, Red, Green, and Red-edge) of light spectrum. Under optimum conditions, healthy plants look green because they absorb red bands and reflect green band of light spectrum (Hatfield et al., 2008). Strong relationship of these combination of light has been reported with photosynthesis, stress and nutrient status in plants (Hatfield et al., 2008; Shafian et al., 2018). For example, normalized difference vegetation index (NDVI), red edge chlorophyll index (CIRed-edge), normalized difference red-edge (NDRE) have been used to differentiate genotypes for stay-green, water stress, growth under N-fertilizer and chlorophyll level (Potgieter et al., 2017; Li et al., 2018; Zheng et al., 2018; Zhang and Zhou, 2019). Grain yield has also been predicted through UAV-based sensors in wheat and sorghum (Han et al., 2018; Hassan et al., 2018b). Therefore, multispectral vegetation indices can be used to assess the status of water and nitrogen and their fluctuations under diverse environmental conditions. The aims of this study were to (1) assess UAV-based multispectral platform determining water and N use efficiencies, (2) evaluate the water and N-fertilizer application strategy using UAV, and (3) identify the genotypes for high water and N efficiency.

## Materials and Methods

### Germplasm and Experimental Design

Three cultivars Zhongmai 895, Aikang 58, and Zhoumai 18 were used to evaluate the accuracy of UAV-based multispectral imagery to predict effectiveness of water and N-fertilizer dosage as well as the potential of genotypes for their uptake efficiencies. These cultivars were released in the Yellow and Huai River Valleys Winter Wheat Region of China over the last decades. The study panel have been known as most prominent varieties for drought resistance and yield potential across the cultivated area by performing differently in stay-green during extreme drought and high temperatures.

**FIGURE 1:**
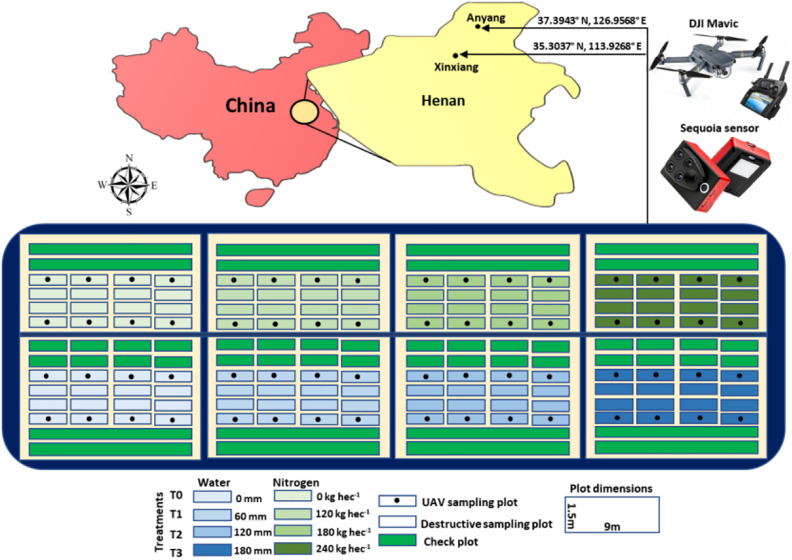
Trial locations, experimental design and UAV platform for phenotyping of water and N use efficiencies.

Two field trials (water and nitrogen) were conducted at two sites i.e., Anyang (37.3943°N, 126.9568°E) and Xinxiang (35.3037°N, 113.9268°E) in Henan province during 2016–2018. Both trials were consisted of two types of experimental plots (a) destructive sampling plots following CIMMYT manual and (b) non-destructive phenotyping plots using UAV platform across the treatments (Figure 1). Randomized complete blocks with three replications each for destructive and UAV based phenotyping were used to minimize the probability of experimental error. Each experimental plot consisted of 6 rows of 9 m in length, 1.5 m in width, and with 0.2 m inter-row spacing. Seeds were planted with a seedling rate of 270 seedlings/m^2^ at both sites. In water use efficiency trial, each replication comprised four water levels, viz. zero (control) T0 = 0, T1 = 60 mm, T2 = 120 mm, and T3 = 180 mm at both sites (Figure 1). Nutrients level for water treatments was maintained at the optimal level. While N use efficiency experiment was also consisted of four levels of N for each three replications viz. zero (control) T0 = 0, T1 = 120 kg ha^–1^, T2 = 180 kg ha^–1^, and T3 = 240 kg ha^–1^. Harvesting was done through combine harvester after full maturity to estimate grain yield.

### UAV-Based Platform and Flight Campaigns

A Mavic Pro (SZ DJI Technology Co., Shenzhen, China) carrying sequoia 4.0 multi-spectral sensor (Micasense Parrot, Seattle, WA, United States)^1^ was used for multispectral imagery over the trials. Mavic pro can fly with slow speed and low altitude for 18 min. Multispectral sensor consists of 4 spectral bands (green, red, red-edge and nir) and a sunshine sensor connected with multi-spectral sensor was installed on the top of UAV to measure environmental irradiation and post-calibrate reflectance. A standardization of band values before and after flight was done through a calibration board with known reflectance. Altizure DJI version 3.6.0^2^ was used to design the flight mission over the trials. All the flights were conducted at 30 m altitude with 2.5 m/s speed, maintaining 85% forward and side overlaps among images. Average ground sample distance of sensor was recorded 2.5 cm. UAV-based multispectral data were collected at heading, flowering and three times at early, mid and late grain filling stages. Data captured at early to late grain filling were averaged to get overall status of genotypes at maturity stage (Table 1).

**TABLE 1:** Multi-spectral indices, their function and data acquisition schedule for both experiments.

Traits		Equation	Function
RNDVI	Red normalized difference vegetation index	RNDVI = (RNIR - Rred)/(RNIR + Rred)	N contents, Biomass
GNDVI	Green normalized difference vegetation index	GNDVI = (RNIR - Rgreen)/(RNIR + Rgreen)	Green biomass, Chlorophyll
NDRE	Normalized difference red-edge index	NDRE = (RNIR - Rred-edge)/(RNIR + Rred-edge)	Greenness
RECI	Red-edge chlorophyll index	NDRE = (RNIR/Rred-edge)-1	Chlorophyll contents
NGRDI	Normalized green red difference index	NGRDI = (Rgreen – Rred)/(Rgreen + Rred)	Yellowing

**Schedule**			

**Stage**	**No. of flights**	**Data calculation**	**Ground data**

Heading (H) (ZS-56)	3	Average (H)	Dry biomass (m^–1^)
Flowering (F) (ZS-66)	3	Average (F)	Dry biomass (m^–1^)
Early grain filling (EGF) (ZS-74)	3	Average of grain filling stages	Dry biomass (m^–1^), Grain yield
Mid grain filling (MGF) (ZS-84)	3		
Late grain gilling (LGF) (ZS-90)	3		

### Image Processing and Data Extraction

All images captured from Sequoia contained accurate geo-referencing due to its built-in GPS device. GPS information was accurate enough to generate dense point cloud for good quality orthomosaic. Pix4D mapper (Version 1.4, PIX4d, Lausanne, Switzerland)^3^ was used for orthomosaic generation. The key steps of the orthomosaic generation using Pix4D mapper comprised camera alignment, geo-referencing, building point clouds and orthomosaic generation as previously reported (Hassan et al., 2018a, b). QGIS was used for image segmentation to extract the useful information of each plot. For this, polygon shapes were generated with a specific plot ID defining the particular germplasm (Haghighattalab et al., 2016). Spectral values were analyzed using combined orthomosaic TIFF images contain four different bands and polygon shape files in IDL (Version 8.6, Harris, Geospatial Solutions, Inc. Reston, CO, Australia, United States). Reflectance calibration was done using calibrated reflectance panel with known reflectance values provided by Micasense (Micasense Parrot, Seattle, WA, United States). Multispectral images of reflectance panel were captured before and after each flight to calibrate the reflectance maps of each stage.

### Estimation of Multi-Spectral Vegetation Indices

Five multispectral traits i.e., red normalized difference vegetation index (RNDVI), green normalized difference vegetation index (GNDVI), normalized difference red-edge (NDRE), red-edge chlorophyll index (RECI), and normalized green red difference index (NGRDI) were also calculated which mainly surrogate to canopy physiological traits including green biomass, chlorophyll level and photosynthesis (Table 1). These indices were calculated from reflectance captured during multi-spectral imagery through Sequoia 4.0 sensor. The calculated multispectral traits were evaluated for non-destructive assessment of water and nitrogen use efficiency by considering cost-effective replacement of traditional destructive indicator such as biomass, N-contents and chlorophyll level under different water and N supply.

### Estimation of Soil Moisture, N Contents, Biomass and Yield Related Traits

The volumetric soil water content of the planting zone was measured for every 10 cm section of soil, down to 160 cm using a CNC503D neutron moisture meter (Super Energy. Nuclear Technology Ltd., Beijing, China). The water content of the soil surface of around 20 cm was also measured using the oven-drying method to minimize the error probability in calculating the WUE. Soil was oven-dried at 105°C until a consistent weight, and then pre-and post-drying weights were compared to determine the water content. This measurement was repeated after all irrigation treatments and major precipitation events. Both methods (volumetric measurements and oven-drying) were used to measure total soil moisture as described by Ma et al. (2016).

For soil N content, soil sampling was done from each subplot at the depth of 0–20, 20–40, 40–60, 60–80, and 80–100 cm for the estimation of before sowing soil N contents. Samples were digested, and modified Kjeldahl method was used to determine total N contents in soil samples as described in Bremner and Mulvaney (1982). For plant N contents at flowering and maturity, samples from each subplot was taken by cutting 20 fertile shoots at ground level. Then, shoots were divided into leaves, straw, sheath at flowering and grains at maturity. Total N content was calculated from above mention methodology. Dry biomass was calculated at heading, flowering and maturity stages by destructive method, while yield related traits including spike number (SN), thousand grain weight (TGW), and grain yield (GY) were calculated as described in Gao et al. (2017).

### WUE and NUE Measurement and Statistical Analysis

Water use efficiency (WUE) from grain yield and dry biomass were calculated using following formulas by Zhang et al. (2007),

WUE.GY=GY/ET

and

WUE.BM=BM/ET

Where, GY is grain yield, Y (kg ⋅ ha^–1^) is genotypes grain yield, BM (kg ⋅ ha^–1^) is dry biomass at maturity and ET (mm) is the evapotranspiration during the winter wheat growing seasons. ET was estimated as follows (Eberbach and Pala, 2005).

ET=I+P-R-D-ΔS

Where, I (mm) is the irrigation water amount; P (mm) is precipitation, which was measured at the on-site weather station using a standard rain gauge given in Supplementary Table S1; R (mm) is the surface runoff, which was assumed as non-significant since concrete slabs were placed around each plot; D (mm) is the downward flux below the crop root zone, which was defined as insignificant since soil moisture measurements indicated that drainage at the sites were negligible; and ΔS (mm) is the change in water storage in the soil profile that is exploited by crop roots (initial soil water content minus soil water content at the end of the growing season).

NUE efficiency was measured using following formulas as demonstrated in Haile et al. (2012),

$$\mathrm{NUE}.\mathrm{GY}={\mathrm{GDW}}_{f}-{\mathrm{GDW}}_{c}/N_{s}$$

and

$$\mathrm{NUE}.\mathrm{NC}=N_{\mathrm{tf}}-N_{\mathrm{tc}}/N_{s}$$

Whereas, GDWf is grain dry weight in fertilized treatment, GDWc is grain dry weight under control treatment while Ns means total N supply in a particular treatment. Ntf indicates total N content in above ground plant sample in fertilized treatment and Ntc means N concentration in plant sample in control treatment.

Pearson correlation and linear regression between the traits were calculated to check relationship and prediction. Significance variation test at *P* < 0.05 and performance of genotypes for traits under different water and N treatments were determined by analysis of variance (ANOVA) using R package (R Core Team, 2016). While repeatability of UAV based data was also calculated to ensure the accuracy by following the formula reported in Sehgal et al. (2015).

## Results

### Correlations of UAV-Based Traits With Indicators of Water Status and WUE

**FIGURE 2:**
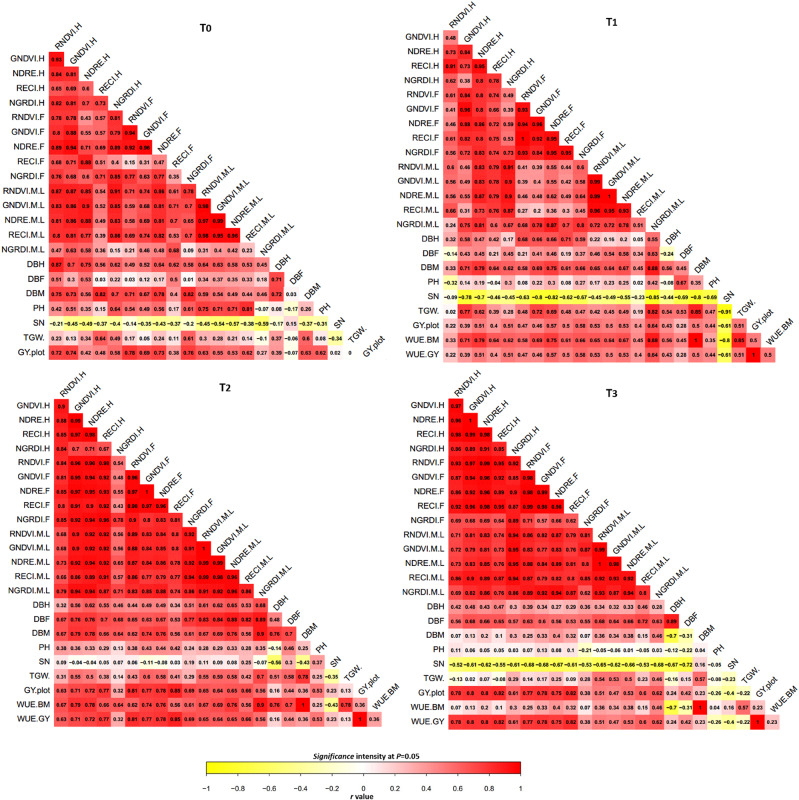
Correlation map between traits under four water treatments across the two experimental sites. Red and yellow colors indicating intensity of r values from positive to negative. T, treatment; H, heading; F, flowering; M.LGF, mid to late grain filling; GNDVI, green normalized difference vegetation index; NDRE, Normalized difference red-edge; RECI, red-edge chlorophyll index; NGRDI, normalized green red difference index; TGW, thousand grain weight; DBH, dry biomass at heading; DBF, dry biomass at flowering; DBM, dry biomass at maturity; SN, spike number per; GY.plot, grain yield per plot; WUE.GY and BM, water use efficiency calculated from grain yield and biomass.

All multispectral traits i.e., red normalized difference vegetation index (RNDVI), green normalized difference vegetation index (GNDVI), normalized difference red-edge (NDRE), red-edge chlorophyll index (RECI), and normalized green red difference index (NGRDI) mainly surrogate to green biomass, water content and chlorophyll level of plants were positively correlated with manually measured indicators of water status i.e., biomass, temporally at heading (*r* = 0.17 to 0.87), flowering (*r* = 0.15 to 0.77) and maturity (*r* = 0.15 to 0.90) in control and all water-supply treatments (Figure 2). Weak to strong correlations between UAV-based multispectral traits with thousand grain weight (TGW) (*r* = 0.14 to 0.84), and moderate to high with grain yield (GY) (*r* = 0.27 to 0.65) were observed across the treatments at mid to late grain filling stage. Plant height showed positive correlations (*r* = 0.17 to 0.81) with RNDVI, GNDVI, NDRE, and RECI in T0, T1 and T2. Water use efficiency calculated from GY (WUE.GY) was strongly correlated with RNDVI (*r* = 0.51 to 0.65), GNDVI (*r* = 0.47 to 0.64), NDRE (*r* = 0.53 to 0.65), RECI (*r* = 0.40 to 0.66), and NGRDI (*r* = 0.56 to 0.64). While water use efficiency estimated from biomass (WUE.BM) also exhibited similar trend in correlation with RNDVI (*r* = 0.36 to 0.67), GNDVI (*r* = 0.34 to 0.69), NDRE (*r* = 0.38 to 0.76), RECI (*r* = 0.15 to 0.56), and NGRDI (*r* = 0.46 to 0.90) across the water-supply treatments. Correlations between multispectral traits and WUE.BM were slightly lower in T3 compared to T1 and T2 treatments. UAV-based multispectral traits were also positively associated with both NUE at heading (*r* = 0.10 to 0.82) and flowering (*r* = 0.25 to 0.85) (Figure 2). Weak but positive correlations were also observed between ground based WUE indicator (biomass) and WUE.GY ranging from *r* = 0.23 to 0.50 at mid to late grain filling stage across the water-supply treatments.

### UAV-Based Prediction of WUE and Variations in Genotypes

Significant variations (*P* < 0.05) among the genotypes and in multispectral traits at mid to late grain filling stage were calculated, with high repeatability ranging from 0.78 to 0.89 (Table 2). In our results, similar trend in variation among WUE and multispectral traits at mid to late grain filling stage were observed that was influencing the GY. Variation results were also validated through ground based destructive measurements (Table 2).

**TABLE 2:** Significance test and repeatability of traits for three cultivars under four water treatments and two locations.

Traits	Cultivars			Treatments				Locations		Data repeatability	
	C895	C58	C18	T0	T1	T2	T3	Xinxiang	Anyang	Xinxiang	Anyang
RNDVI.H	0.63a	0.62a	0.60b	0.54d	0.61c	0.65b	0.67a	0.61a	0.63a	0.79	0.81
GNDVI.H	0.53a	0.50b	0.48c	0.44d	0.51c	0.53b	0.55a	0.49b	0.53a	0.86	0.84
NDRE.H	0.23a	0.19b	0.17c	0.14c	0.20b	0.21b	0.23a	0.29a	0.20b	0.85	0.87
RECI.H	0.60a	0.53b	0.44c	0.33c	0.53b	0.56b	0.68a	0.83a	0.82a	0.78	0.86
NGRDI.H	0.19a	0.18b	0.17c	0.15d	0.162c	0.20b	0.21a	0.20a	0.16b	0.8	0.78
RNDVI.F	0.58a	0.56b	0.55b	0.48c	0.55b	0.60b	0.61a	0.58a	0.54b	0.78	0.79
GNDVI.F	0.47a	0.44b	0.43b	0.38d	0.44c	0.47b	0.49a	0.46a	0.43a	0.84	0.83
NDRE.F	0.23a	0.19b	0.17c	0.143d	0.19c	0.22b	0.24a	0.29a	0.14b	0.85	0.89
RECI.F	0.59a	0.52b	0.47c	0.33d	0.49c	0.58b	0.69a	0.74a	0.62b	0.78	0.72
NGRDI.F	0.17a	0.16b	0.15c	0.12c	0.15b	0.18a	0.18a	0.16a	0.16b	0.89	0.85
RNDVI.M-L	0.36a	0.35a	0.31b	0.20c	0.31b	0.44a	0.44a	0.45a	0.33b	0.87	0.86
GNDVI.M-L	0.35a	0.33b	0.30c	0.24c	0.33cb	0.38a	0.39a	0.36a	0.29b	0.79	0.89
NDRE.M-L	0.163a	0.12b	0.11b	0.084c	0.12b	0.164a	0.169a	0.16a	0.10b	0.86	0.87
RECI.M-L	0.28a	0.23b	0.20c	0.10c	0.21b	0.33a	0.34a	0.42a	0.35b	0.88	0.87
NGRDI.M-L	0.12a	0.10b	0.09c	0.04c	0.078b	0.15a	0.15a	0.12a	0.09b	0.87	0.83
DBH (g/m)	136.47b	140.28a	139.67a	112.95d	126.29c	142.05b	173.93a	118.31b	159.30a	0.79	0.83
DBF (g/m)	205.12a	198.13b	197.71b	170.710c	184.97b	219.34a	220.93a	189.76b	208.21a	0.8	0.78
DBM (g/m)	282.38a	270.67b	270.15b	185.05c	238.11b	334.93a	339.51a	322.98a	295.82b	0.83	0.79
SN	23.74b	27.53a	27.00a	22.01b	28.30a	26.89a	27.164a	26.244a	25.94b	0.78	0.78
PH (cm)	72.138a	70.21b	70.43b	70.73a	70.54a	70.77a	71.67a	70.96a	70.89a	0.86	0.88
TGW (g)	50.38a	44.12c	45.59b	45.30c	46.47b	47.12ab	47.89a	49.16a	44.23b	0.79	0.78
GY Kg/plot	7.23a	7.17a	6.36b	4.53c	5.79b	6.71a	6.95a	6.36a	5.59b	0.8	0.79
WUE.BM Kg/hec/mm	55.17a	52.52b	52.42b	–	51.19c	61.13b	62.87ab	71.32a	55.42b	0.82	0.81
WUE.GY Kg/hec/mm	1.59a	1.58a	1.50b	–	1.57b	1.61a	1.65a	1.92a	1.54b	0.83	0.8

*Alphabets are indicating significance differences at P > 0.05 level. C895; Zhongmai 895, C58; Aikang 58 and C18; Zhoumai18. T, treatment; H, heading; F, flowering; M.LGF, mid to late grain filling; GNDVI, green normalized difference vegetation index; NDRE, Normalized difference red-edge; RECI, red-edge chlorophyll index; NGRDI, normalized green red difference index; TGW, thousand grain weight; DBH, dry biomass at heading; DBF, dry biomass at flowering; DBM, dry biomass at maturity; SN, spike number per; GY, grain yield; WUE.GY and BM, water use efficiency calculated from grain yield and biomass.*

**FIGURE 3:**
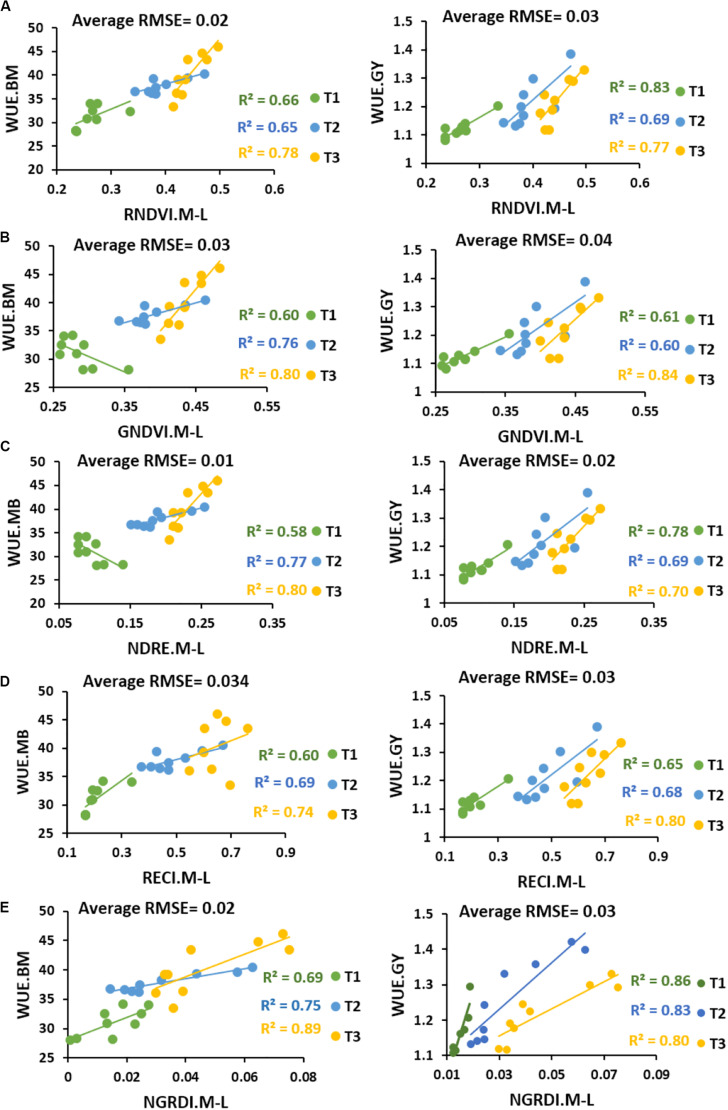
Coefficients of determination results between multispectral traits **(A)** RNDVI, **(B)** GNDVI, **(C)** NDRE, **(D)** RECI, and **(E)** NGRDI and WUE calculated from biomass (WUE.BM) and estimated from grain yield (WUE.GY) under water-supply treatments.

**FIGURE 4:**
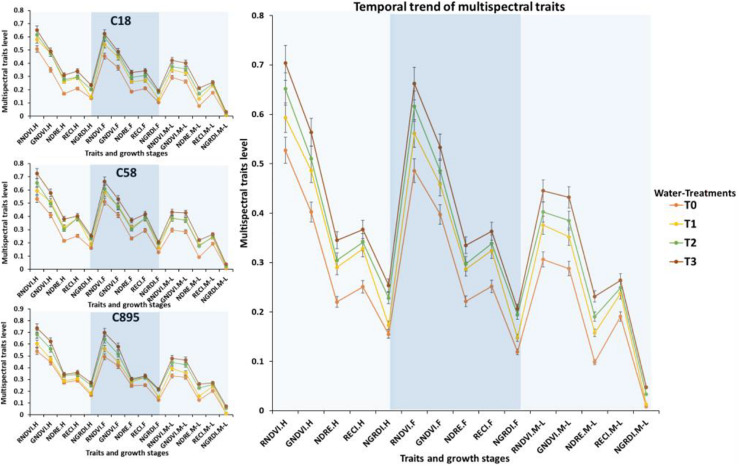
Seasonal trend in multispectral traits and comparison of genotypes under water treatments. Error bars are showing standard deviation. *C895, Zhongmai 895; C58, Aikang 58; C18, Zhoumai18.

Linear regression model was fitted to forecast both WUE.BM and WUE.GY through UAV-based multispectral traits (Figure 3). Results showed high *R*^2^ values for RNDVI (*R*^2^ = 0.65 to 0.83), GNDVI (*R*^2^ = 0.60 to 0.84), NDRE (*R*^2^ = 0.58 to 0.80), RECI (*R*^2^ = 0.60 to 0.80) and NGRDI (*R*^2^ = 0.69 to 0.89) in predicting both WUE at mid to late grain filling stage, while average root mean square errors (RMSE) ranged from 0.002 to 0.04. NGRDI traits was found better with higher coefficients of determination values in predicting both WUE.BM (*R*^2^ = 0.69 in T1, *R*^2^ = 0.75 in T2 and *R*^2^ = 0.89 in T3) and WUE.GY (*R*^2^ = 0.86 in T1, *R*^2^ = 0.83 in T2 and *R*^2^ = 0.80 in T3) with low average RMSE 0.02 and 0.03. Whereas, UAV-based traits also predicted significant differences (*P* < 0.05) between the water treatments and locations. In Figure 4, fluctuation in dynamic trend of multispectral traits values in all four treatments high for Zhongmai 895 compare to other two genotypes. Dynamic curves showed low level in multispectral traits at mid to late grain filling stage under control treatment. Zhongmai 895 also performed high in both WUE under water-supply treatments, but there was no significant difference in WUE and GY between T2 and T3 (Table 2).

### Correlations of UAV-Based Traits With Ground Indicators of N-Status and NUE

**FIGURE 5:**
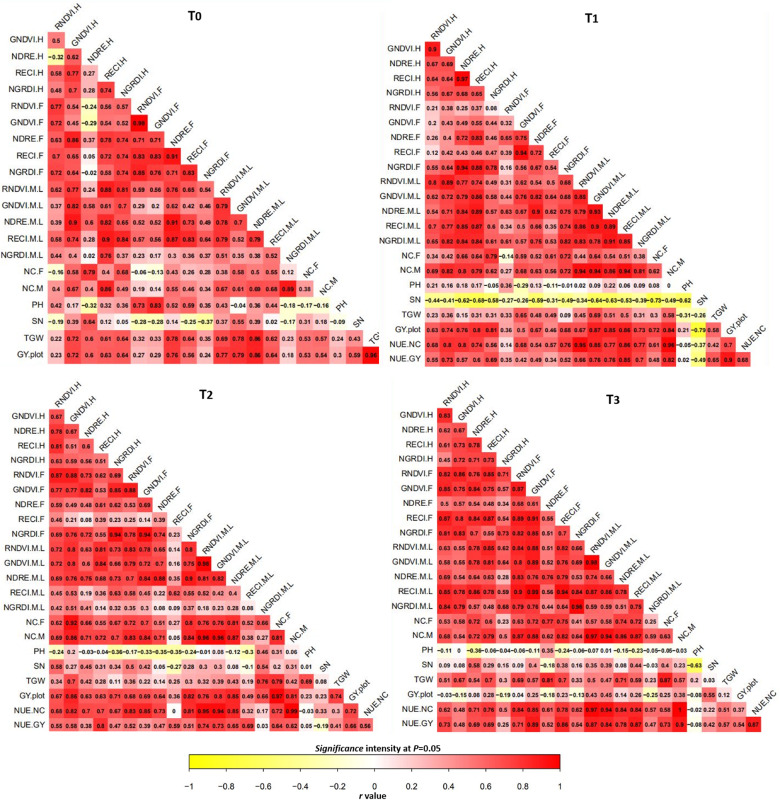
Correlation map between traits under four nitrogen treatments across the two experimental sites. Red and yellow colors indicating intensity of r values from positive to negative. T, treatment; H, heading; F, flowering; M.LGF, mid to late grain filling; GNDVI, green normalized difference vegetation index; NDRE, Normalized difference red-edge; RECI, red-edge chlorophyll index; NGRDI, normalized green red difference index; TGW, thousand grain weight; DBH, dry biomass at heading; DBF, dry biomass at flowering; DBM, dry biomass at maturity; SN, spike number per; GY.plot, grain yield per plot; NUE.GY and NC, water use efficiency calculated from grain yield and nitrogen contents.

In Figure 5, UAV-based five multispectral traits were positively correlated with N-contents ranging from *r* = 0.27 to 0.84 at flowering and *r* = 0.27 to 0.97 at maturity in in all N-supply treatments. NGRDI showed quite low correlations with N-contents at both flowering and maturity in T2 and T3 compared with T0 and T1 where r-values were high ranging from 0.81 to 0.89 (Figure 5). A consistently moderate to high correlations of multispectral traits with TGW ranging from *r* = 0.23 to 0.86 and GY *r* = 0.49 to 0.86 were noticed at the maturity stage. There were negative to positive correlations (*r* = −0.26 to 0.46) between plant height, multispectral traits and N-contents across the treatments. NUE.NC calculated from nitrogen contents of plant samples at maturity was correlated with all five remotely sensed multispectral traits at *r* = 0.23 to 0.97, while GY based nitrogen use efficiency (NUE.GY) at *r* = 0.47 to 0.87 across the treatments. Similar correlations trend ranging from *r* = 0.23 to 0.90 were also observed between ground based NUE indicator (N-contents) and NUE.NC in T2 and T3, and with NUE.GY ranging from *r* = 0.27 to 0.99 at maturity stage across the treatments (Figure 5). Significantly low to strong correlations were also detected between NUE and UAV-based multispectral traits at heading (*r* = 0.38 to 0.82) and flowering (*r* = 0.14 to 0.89).

### UAV-Based Prediction of NUE and Variations in Genotypes

**FIGURE 6:**
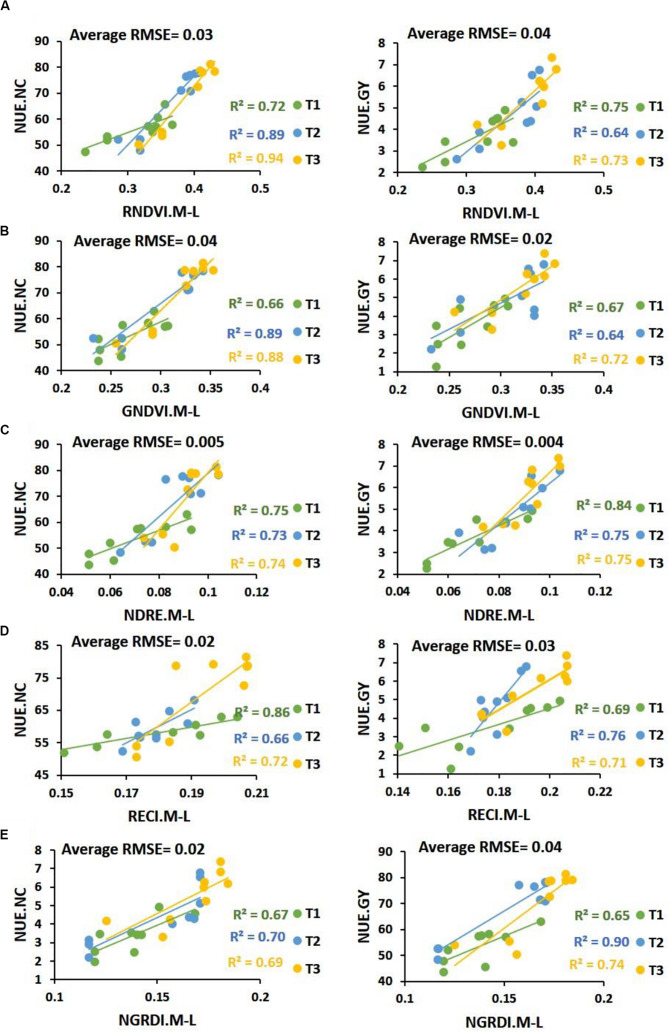
Coefficient of determination results between multispectral traits **(A)** RNDVI, **(B)** GNDVI, **(C)** NDRE, **(D)** RECI, and **(E)** NGRDI and NUE calculated from plant nitrogen contents NUE.NC and estimated from grain yield NUE.GY under N-supply treatments.

High coefficients of determination were identified between UAV-based traits and NUE calculated from nitrogen content of plant samples (NUE.NC) and GY (NUE.GY) with low RMSE ranging from 0.004 to 0.04 across the N-supply treatments (Figure 6). Strong *R*^2^ values of RNDVI (*R*^2^ = 0.64 to 0.94), GNDVI (*R*^2^ = 0.64 to 0.89), NDRE (*R*^2^ = 0.73 to 84), RECI (*R*^2^ = 0.66 to 0.86 and NGRDI (*R*^2^ = 0.65 to 0.90) with NUE.NC and NUE.GY predicted significant variations (*P* < 0.05) among genotypes at mid to late grain filling stage with high repeatability ranging from 0.80 to 0.91 (Table 3). RNDVI was high in predicting NUE.NC in T1 (*R*^2^ = 0.72), T2 (*R*^2^ = 0.89), and T3 (*R*^2^ = 0.94), while NDRE was also found consistent in forecasting both NUE.NC with high *R*^2^ = 0.75 in T1, *R*^2^ = 0.73 in T2, *R*^2^ = 0.74 in T3 and NUE.GY by *R*^2^ = 0.84 in T1, *R*^2^ = 0.75 in T2 and T3 with RMSE of 0.004 to 0.005.

**TABLE 3:** Significance test and repeatability of traits for three cultivars under four N treatments and two locations.

Traits	Cultivars			Treatments				Locations		Data repeatability	
	C18	C58	C895	T0	T1	T2	T3	Xinxiang	Anyang	Xinxiang	Anyang
RNDVI.H	0.64b	0.67a	0.68a	0.59c	0.65b	0.67b	0.74a	0.66a	0.67a	0.91	0.91
GNDVI.H	0.54b	0.57a	0.59a	0.50c	0.55b	0.58b	0.64a	0.56a	0.59a	0.86	0.84
NDRE.H	0.17b	0.24b	0.26a	0.19c	0.20c	0.24b	0.27a	0.22a	0.23a	0.85	0.89
RECI.H	0.52b	0.58a	0.60a	0.52b	0.53b	0.59a	0.60a	0.56a	0.58a	0.8	0.82
NGRDI.H	0.18ab	0.19a	0.20a	0.16c	0.17c	0.20b	0.24a	0.19a	0.21a	0.8	0.81
RNDVI.F	0.54ab	0.56a	0.57a	0.49c	0.55b	0.59a	0.61a	0.55a	0.6a	0.83	0.79
GNDVI.F	0.43b	0.45a	0.47a	0.37c	0.44b	0.49a	0.51a	0.45b	0.53a	0.84	0.83
NDRE.F	0.15b	0.19a	0.20a	0.10c	0.16b	0.20a	0.21a	0.18b	0.22a	0.85	0.9
RECI.F	0.31b	0.39a	0.42a	0.27c	0.33b	0.43a	0.44a	0.37b	0.41a	0.8	0.87
NGRDI.F	0.15ab	0.16a	0.16a	0.14c	0.17b	0.19a	0.19a	0.15b	0.22a	0.83	0.91
RNDVI.M-L	0.29c	0.32b	0.37a	0.26c	0.31b	0.36a	0.39a	0.32a	0.35a	0.9	0.86
GNDVI.M-L	0.24bc	0.27b	0.31a	0.23c	0.26b	0.30a	0.31a	0.273a	0.28a	0.91	0.85
NDRE.M-L	0.05bc	0.07ab	0.09a	0.03c	0.09b	0.11a	0.13a	0.07a	0.1a	0.82	0.81
RECI.M-L	0.12c	0.19b	0.23a	0.10c	0.17b	0.21a	0.23a	0.18a	0.2a	0.85	0.91
NGRDI.M-L	0.10bc	0.11b	0.13a	0.09c	0.16b	0.18a	0.19a	0.11a	0.13a	0.83	0.8
NC.F (mg)	201.73b	210.52a	216.94a	195.16c	205.58b	216.96a	215.89a	209.73a	208.8a	0.8	0.87
NC.M (mg)	45.90c	51.56b	57.32a	40.00d	47.88c	56.15ab	62.36a	51.59a	53.56a	0.8	0.8
PH (cm)	66.50a	66.28	67.41a	66.66a	64.78a	66.70a	68.78a	66.72a	69.45a	0.9	0.83
SN	32.89a	33.06a	32.76a	32.41a	34.33a	33.387a	31.48a	32.90a	33.23a	0.86	0.87
TGW (g)	48.06b	47.89b	53.20a	46.66c	49.21ab	51.20a	53.81a	49.71a	50.4a	0.85	0.93
GY (Kg/plot)	11.00b	11.39b	12.40a	10.11c	11.0b	11.88a	11.91a	11.59a	11.9a	0.78	0.86
NUE.NC (kghec^–1^)	50.71c	63.61b	71.36a	–	53.54b	67.28a	69.86a	61.89a	63.56a	0.83	0.81
NUE.GY (mg1g ^–1^ sample/KgN)	3.24c	4.86b	5.73a	–	3.50c	4.81ab	5.50a	4.61a	4.9a	0.9	0.88

*Alphabets are indicating significance differences at P > 0.05 level. C895; Zhongmai 895, C58; Aikang 58 and C18; Zhoumai18. T, treatment; H, heading; F, flowering; M.LGF, mid to late grain filling; GNDVI, green normalized difference vegetation index; NDRE, Normalized difference red-edge; RECI, red-edge chlorophyll index; NGRDI, normalized green red difference index; TGW, thousand grain weight; SN, spike number per; GY, grain yield; NUE.GY and NC, water use efficiency calculated from grain yield and nitrogen contents.*

**FIGURE 7:**
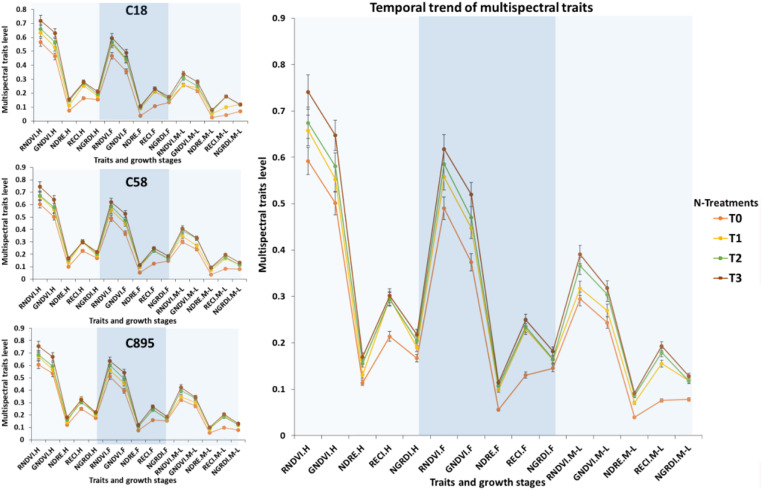
Seasonal fluctuation trend in multispectral traits and comparison of genotypes under N treatments. Error bars are showing standard deviation. *C895, Zhongmai 895; C58, Aikang 58; C18, Zhoumai18.

Traditionally validated indicators of N-status such as N-contents in plant body significantly varied among the three genotypes (*P* < 0.05) at mid to late grain filling stage in all N-supply treatments (Table 3). Similar trend was observed for UAV-based remotely sensed traits, illustrating the variations in NUE and fluctuations in GY among the genotypes and differences between the treatments. Multispectral traits were found significantly higher at flowering and mid to late grain filling stage in Zhongmai 895 compared to other genotypes across the treatments (Table 3 and Figure 7). T2 was the most resourceful in both water and N-supply treatment for resource-effective GY. High curves points for multispectral traits indicated greater N-content, chlorophyll level under water and N-supply, which mean high NUE. Whereas, declining curves of UAV-based traits showed their low level in control treatment at mid to late grain filling stage.

**FIGURE 8:**
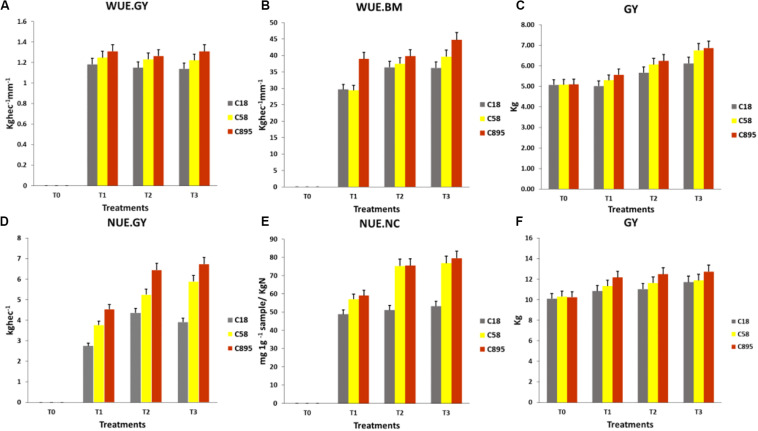
Comparison of genotypes for **(A)** WUE.GY, **(B)** WUE.BM, **(C)** GY under water treatments and **(D)** NUE.GY, **(E)** NUE.NC, **(F)** GY under N-supply treatments. *C895, Zhongmai 895; C58, Aikang 58; C18, Zhoumai18.

Results showed that Zhongmai 895 was elite in NUE.GY compared to Aikang 58 and Zhoumai 18, but in NUE.NC both Zhongmai 895 and Aikang 58 were equally high under T1 and T2 (Figure 8). Whereas, GY of Zhongmai 895 was also higher across the water and N supply treatments compared to control treatment where no yield difference was observed among the three genotypes.

## Discussion

### Potential of UAV-Based Multispectral Traits to Assess Water and N Use Efficiencies

Manual phenotyping is time consuming, expensive and error prone, and should be replaced by advanced, rapid and accurate technology to assess dynamic biomass and N status to describe WUE and NUE (Zhang and Zhou, 2019). Therefore, it is important to establish usefulness of UAV-based remote sensing over the traditional phenotyping approaches. Previously, some studies have predicted physiological status such as senescence rate and grain yield effectively through UAV-based multispectral data in wheat (Duan et al., 2017; Hassan et al., 2018a, b). In our study, strong correlations of UAV-based multispectral traits with traditionally measured indicators of water (biomass) and N status (N contents) in all treatments had validated the UAV-based data for accurate assessments of WUE and NUE (Figures 2, 5). Whereas, higher correlations of UAV-based multispectral traits with WUE and NUE compared to ground-based destructive data had proven its superiority. Similar trend in correlations of multispectral traits, WUE and NUE with GY also indicated the practicality of UAV for selection in wheat breeding. Moreover, high prediction results of UAV-based multispectral traits ranging from *R*^2^ = 0.58 to 0.94 with low RMSE for WUE and NUE using linear regression model, had also exhibited that UAV remote sensing could be capable in detecting within season water and N utilization efficiencies of plant efficiently (Figures 3, 6). Therefore, addition of UAV-based non-destructive phenotyping platform to large breeding programs could be better replacement of traditional approaches and helpful to reduce the cost of labor and time in assessing biomass, N contents and resource efficiencies in wheat.

### Comparison of Multispectral Traits in Response to Water and N-Supply

Visible sign of efficient supply of water and N-fertilizer are high greenness, chlorophyll and N level in plant body. Spectral bands have strong relationship with physiological indicators of nutrient’s status such as green cover, chlorophyll, N contents (Li et al., 2018) and can demonstrate the water stress severity and N status in plants (Wang et al., 2013; Zheng et al., 2018). But due to different features of spectral bands, some limitations have been reported in precise physiological information at particular growth stages (Hatfield et al., 2008). For example, it is reported that red band is more reliable at pre-maturation stages due to saturation issues in detecting high chlorophyll level after canopy closure. Therefore, it makes difficult to sense minor variations in spectral indicators of plants physiology. Near infrared (NIR) band is strongest in detecting long range of variations in green biomass and N-status. While reflectance in the green and the red-edge bands ranges are also sensitive to the whole range of variations in chlorophyll and green biomass (Hatfield et al., 2008; Li et al., 2018).

In water trial, multispectral traits derived from NIR, red and green bands (RNDVI, GNDVI and NGRDI) had shown strong relationship with temporally measured biomass, WUE and GY. Especially NGRDI has shown low to high correlations (*r* = 0.27 to 0.90) with WUE and determined the significant (*P* < 0.05) variations among three wheat genotypes at mid to late grain filling stage in water-supply treatments (T1, T2, and T3) (Figure 2 and Table 2). Whereas, high repeatability of UAV-based multispectral traits indicated that these traits could be reliable for prediction of WUE (Table 2). Similar correlation results of RNDVI and NGRDI have also been reported for efficient prediction of biomass and GY under different water and N conditions (Duan et al., 2017; Hassan et al., 2018b). Low correlations with GY in control treatment (T0) might be due to saturation in red band after early canopy closure under drought severity, while low greenness could also cause low reflectance of green band. Under normal condition, healthy plant showed high reflectance of NIR band, and low red and re-edge bands. Whereas, green, red and red-edge bands showed high reflectance as compare to NIR under water stress. NGDRI was derived from subtracting red from the green band, which means calculating the fraction of reflected red band from the visible green band. This fractional information about the red band could provide deep information about minor proportion of yellowing in plants (Mutanga and Skidmore, 2004; Hatfield et al., 2008). Therefore, NGDRI could be useful to detect greenness and health of plants under normal growing condition. Whereas, GNDVI derived from NIR and green band was highly correlated (up to *r* = 0.70) with biomass, at heading to flowering stages compared to mid to late grain filling stage, indicating that this trait could predict the water status at pre-maturation stages effectively. Moderate to strong correlations ranging from *r* = 0.33 to 0.82 of NDRE and RECI traits derived from NIR and red-edge band with green biomass at particular stage and WUE were also recorded efficient to detect variations among the genotypes across the treatments (Figure 2 and Table 2). Red-edge band has been reported to detect water stress severity because it could cover wider range of chlorophyll level as compare to red band (Sharma et al., 2015). Therefore, despite of similar trend in correlation results, NDRE and RECI could be more reliable traits compared to RNDVI and GNDVI to predict the biomass, WUE and variations among the genotypes at mid to late grain filling stage due to known advantage of red-edge band over red band (Mutanga and Skidmore, 2004).

In N-supply experiment, moderate to strong correlations of all multispectral traits with biomass, N-contents, NUE and GY across the growth stages indicated that UAV-based phenotyping can also be useful in selection of N-efficient genotypes and the assessment of crop cultivation method. Despite the previous finding that biomass varied greatly at early growth stages which can mask the effect of N-supply (Haboudane et al., 2002), our results had shown moderate to strong correlations ranging from *r* = 0.27 to 0.84 and *r* = 0.27 to 0.97 between multispectral traits and N-contents, respectively, at heading and flowering stages. Moreover, Haboudane et al. (2002) had reported weak capability of UAV-based spectral indices in assessing N-status after heading because of structural constraints of canopy that cause saturation in reflectance of bands (Haboudane et al., 2002). But in our results, UAV-based multispectral traits showed high ability to detect N-status and NUE at mid to late grain filling stage with high r values [up to 0.97 (Figure 5)]. Interestingly, RNDVI, GNDVI, NDRE, and RECI performed equally in estimating N-contents and NUE at mid to late grain filling stages. RNDVI and NDRE showed high correlations (up to 0.97) with both NUE.NC and NUE.GY at mid to late grain filling stage, because it has strong connection in detecting N-status while reflectance of NIR band has been reported higher under application N-fertilizer. Significant variations at (*P* < 0.05) among the genotypes for N-contents and NUE were also successfully assessed through variation in UAV-based multispectral traits effectively with high repeatability across the growth stages and N treatments (Table 3). Whereas, similar correlation trends among destructive measurements of N-content and multispectral traits for NUE, TGW, and GY indicated that UAV based non-destructive sensing could be a cost-effective replacement.

In conclusion, NGDRI was more water-sensitive under water efficient conditions, while NDRE and RECI were drought sensitive under water-deficient conditions. Whereas, RNDVI, GNDVI, NDRE, and RECI showed equally better sensitivity to assess NUE across the growth cycle. Especially, RNDVI and NDRE were consistent in forecasting variations for both NUE.NC and NUE.GY.

### Significance of UAV-Based Prediction of WUE and NUE for Genotypic Selection

Destructive assessment regarding efficiency of water and N-fertilizer application for different genotypes and evaluation of cultivation approaches have remained a bottleneck for resourceful improvement of crop yield (Boschetti et al., 2014; Zheng et al., 2018). It is due to laborious and error prone work across the season in case large number of genotypes which might mislead the agriculturist during selection. Previously, few studies have been conducted in detecting water and nutrient status of plant using non-destructive remote sensing from both ground and aerial platforms (Haile et al., 2012; Li et al., 2018; Zheng et al., 2018; Zhang and Zhou, 2019). But there is no report on practical application of aerial platform to predict the WUE and NUE for evaluating variations in genotypes under different water and N-supply levels. This study had predicted significant variations successfully among the genotypes for water and N uptake efficiencies and their impact on grain yield through UAV-based multispectral traits with high regression values of *R*^2^ = 0.58 to 0.89 and *R*^2^ = 0.64 to 0.94, respectively, with low root mean square error (Figures 3, 6). These results had proven the usefulness of non-destructive aerial phenotyping compared to ground-based assessments with high repeatability. Water and N-fertilizer demand of different genotypes at particular growth stages is varied. Therefore, prediction of slight fluctuation in water and N-status and its impact on biomass development, chlorophyll level through generating UAV-based multispectral dataset could be helpful in accurate selection. In our results, genotype Zhongmai 895 performed significantly better in WUE and NUE for enhancement of GY at mid to late grain filling stages as compared to other two genotypes across the treatments of both experiments (Tables 2, 3). While at this stage, there was also high level of multispectral traits for Zhongmai 895 which indicated the usefulness of UAV-based phenotyping to predict WUE and NUE (Figures 4, 7). Superiority of Zhongmai 895 for NUE can also be validated by recently study Yang et al. (2020), which has reported seedling vigor of Zhongmai 895 cultivar under high N condition. Therefore, these remotely sensed traits could be a rapid and cost-effective replacement of traditional traits for precise selection of genotypes.

### Significance of UAV-Data for Establishing Cultivation Strategy

Resourceful application of water and N-fertilizer is vital to limit huge losses in important resources. For this, development of elite cultivars and efficient supply of water and N-fertilizer could be effective for required high yield from particular genotypes (Guo et al., 2010). In china, availability of irrigation water is decreasing (Zhang et al., 2007), while N resources are also predicted to decrease in coming years (Chuan et al., 2016; Zhang et al., 2016). Whereas, most of the cultivated lands in China is already been reported highly nitrogenous (Chuan et al., 2016). Therefore, appropriate application of water and N could help to cope with these challenges. It is difficult to phenotype large sample size through destructive measurement repeatedly under various water and N-fertilizer regimes. A non-destructive approach in assessing exact requirement for growth of particular genotypes is important factor regarding potential achievement in crop improvement. Our UAV-based results had shown that T1 and T2 were resourceful for WUE and T2 for NUE compared to T3 (Figure 8). There was no significant difference between T2 and T3 in terms of both UAV and ground-based indicator of water and N status as well as in WUE and NUE for GY. But genotypes had shown significant differences in WUE and NUE under T3 in both water and N-supply treatments. It means up-take efficiency of water and N had increased up to T2 for significant enhancement of GY, but T3 was not resourceful in both experiments. Zhongmai 895 was the most resource-efficient genotype across the water and N-supply treatments (Figure 8 and Tables 2, 3). Our result suggested that UAV-based multispectral data could be vital for establishing cultivation strategies for crops.

## Conclusion

In this study, we established that UAV-based remotely sensed multispectral traits could predict the variations among the genotypes for WUE, NUE and their impact on GY. We found that NGDRI was an efficient multispectral trait to detect water status under irrigated conditions, while NDRE and RECI under drought stress. Whereas, RNDVI, GNDVI, NDRE, and RECI were equally better to sense the crop growth in different irrigation regimes in rapid manner. The RNDVI, GNDVI, NDRE, and RECI were equally sensitive for NUE prediction. Especially, RNDVI showed better prediction for NUE.NC, and NDRE was constant in assessing both NUE.NC and NUE.GY. Our results also suggested that T2 in irrigation (120 mm) and N-fertilization (180 kg ha^–1^) trials was the most resource-efficient treatment for all three genotypes, while Zhongmai 895 was elite in WUE and NUE. In future, the inherent mechanism of crop water and N uptake as well as crop morphological and structural properties, coupled with UAV-based remote sensing, will be used to increase the selection accuracy in large breeding programs.

## Data Availability Statement

The datasets generated for this study are available on request to the corresponding author.

## Author Contributions

MY and MH managed the UAV flights for aerial imagery, analyzed the data, and supervised and wrote the manuscript. ZH for supervised the research. MY and KX conducted the ground-based field measurements. YX, YZ, and CZ managed and directed the trial. YX, AR, XX, and XJ gave comments and suggestions during preparation of the manuscript. All authors contributed to the article and approved the submitted version.

## Conflict of Interest

The authors declare that the research was conducted in the absence of any commercial or financial relationships that could be construed as a potential conflict of interest.

## Footnotes

1 https://www.micasense.com/parrotsequoia/

2 https://www.altizure.com

3 https://pix4d.com/

## Supplementary Material

TABLE S1 Total precipitation across the growing season.
