# Supplementary material for: Assessment of Water and Nitrogen Use Efficiencies Through UAV-Based Multispectral Phenotyping in Winter Wheat
Source: Front Plant Sci. 2020 Jun 26;11:927. doi: 10.3389/fpls.2020.00927 (PMC7333459; doi:10.3389/fpls.2020.00927)
Supplement: TABLE S1 — Total precipitation across the growing season. [file Table_1.DOC]

**Assessment of water and nitrogen use efficiencies in bread wheat using UAV-based multispectral phenotyping**

Mengjiao Yang^1, †^, Muhammad Adeel Hassan^1,†^, Kaijie Xu^2^, Chengyan Zheng^1^, Awais Rasheed^1,3,4^, Yong Zhang^1^, Xiuliang Jin^1^, Xianchun Xia^1^, Yonggui Xiao^1,*^ and Zhonghu He^1,4,*^

^1^ Institute of Crop Sciences, National Wheat Improvement Centre, Chinese Academy of Agricultural Sciences (CAAS), Beijing 100081, China

^2^ Institute of Cotton Research, CAAS, 38 Huanghe Dadao, Anyang 455000, Henan Province, China

^3^ Department of Plant Science, Quaid-i-Azam University, Islamabad 44000, Pakistan

^4^ International Maize and Wheat Improvement Centre (CIMMYT) China Office, c/o CAAS, Beijing 100081, China

^†^Mengjiao Yang and Muhammad Adeel Hassan contributed equally to this work.

*Authors for correspondence: Yonggui Xiao, Email: xiaoyonggui@caas.cn, and Zhonghu He, Email: zhhecaas@163.com

**Table S1: Total precipitation across the growing season.**

| **Total Precipitation (mm)** | | | | | | | | | | |
| --- | --- | --- | --- | --- | --- | --- | --- | --- | --- | --- |
| **^Month^/Location** | **Oct-17** | **Nov-17** | **Dec-17** | **Jan-18** | **Feb-18** | **Mar-18** | **Apr-18** | **May-18** | **Jun-18** | **Total** |
| **Xinxiang** | 50.9 | 0.4 | 0.9 | 4.3 | 0.9 | 7.1 | 74.8 | 74.2 | 152.6 | 366.1 |
| **Anyang** | 40.1 | 2.9 | 0.2 | 1.2 | 4.4 | 14.1 | 19.7 | 50.8 | 107.5 | 240.9 |
